# A Virtual Reprise of the Stanley Milgram Obedience Experiments

**DOI:** 10.1371/journal.pone.0000039

**Published:** 2006-12-20

**Authors:** Mel Slater, Angus Antley, Adam Davison, David Swapp, Christoph Guger, Chris Barker, Nancy Pistrang, Maria V. Sanchez-Vives

**Affiliations:** 1 Department of Computer Science, University College London London, United Kingdom; 2 Guger Technologies OEG Schiedlberg, Austria; 3 SubDepartment of Clinical Health Psychology, University College London London, United Kingdom; 4 Instituto de Neurociencias de Alicante, Universidad Miguel Hernandez - Consejo Superior de Investigaciones Científicas Campus de San Juan, San Juan de Alicante, Spain; 5 Institució Catalana de la Recerca i Estudis Avançats ‐ Universitat Politècnica de Catalunya, Virtual Reality Centre of Barcelona Barcelona, Spain; University of Minnesota, United States of America

## Abstract

**Background:**

Stanley Milgram's 1960s experimental findings that people would administer apparently lethal electric shocks to a stranger at the behest of an authority figure remain critical for understanding obedience. Yet, due to the ethical controversy that his experiments ignited, it is nowadays impossible to carry out direct experimental studies in this area. In the study reported in this paper, we have used a similar paradigm to the one used by Milgram within an immersive virtual environment. Our objective has not been the study of obedience in itself, but of the extent to which participants would respond to such an extreme social situation as if it were real in spite of their knowledge that no real events were taking place.

**Methodology:**

Following the style of the original experiments, the participants were invited to administer a series of word association memory tests to the (female) virtual human representing the stranger. When she gave an incorrect answer, the participants were instructed to administer an ‘electric shock’ to her, increasing the voltage each time. She responded with increasing discomfort and protests, eventually demanding termination of the experiment. Of the 34 participants, 23 saw and heard the virtual human, and 11 communicated with her only through a text interface.

**Conclusions:**

Our results show that in spite of the fact that all participants knew for sure that neither the stranger nor the shocks were real, the participants who saw and heard her tended to respond to the situation at the subjective, behavioural and physiological levels as if it were real. This result reopens the door to direct empirical studies of obedience and related extreme social situations, an area of research that is otherwise not open to experimental study for ethical reasons, through the employment of virtual environments.

## Introduction

In an attempt to understand events in which people carry out horrific acts against their fellows Stanley Milgram carried out a series of experiments in the 1960s at Yale University that directly attempted to investigate whether ordinary people might obey the orders of an authority figure to cause pain to a stranger. He showed that in a social structure with recognised lines of authority, ordinary people could be relatively easily persuaded to give what seemed to be even lethal electric shocks to another randomly chosen person [1], [2]. His results are often cited today, for example, recently in helping to explain how people become embroiled in organised prisoner abuse [3] and even suicide bombings [4]. However, his study also ignited a far-reaching debate about the ethics of deception and of putting subjects in a highly distressing situation in the course of research [5], [6], and as a result this line of research is no longer amenable to direct experimental studies.

Milgram's paradigm was an experiment that subjects were led to believe was a study of the effects of punishment on learning. The subjects, referred to as Teachers, were asked to administer electric shocks of increasing voltages to another subject (the Learner) whenever he gave a wrong answer in a word-memory experiment. A lottery to choose who would be ‘Teacher’ and who ‘Learner’ was carried out at the start of the experiment. In fact, the whole situation was contrived: there were no actual shocks, the lottery was fixed, and the Learner was a confederate of the experimenter. Contrary to expectations, a high proportion of subjects (65% in one condition, n = 40) continued to give ‘shocks’ to the maximum 450 volts, in spite of screams of protest from the Learner. Almost all subjects exhibited signs of distress and many expressed their fears regarding the well-being of the Learner, nevertheless continuing to give shocks to the end.

We have carried out a replication of Milgram's experiment, but in an immersive virtual environment, where participants were required to give ‘electric shocks’ – to a virtual human. Our main objective has not been to study obedience but human responses to interaction with a virtual character in the type of extreme social situation exemplified by the conflict created within Milgram's paradigm.

An immersive virtual environment is formed by a computer-generated surrounding real-time (stereoscopic) display of virtual sensory data from a viewpoint determined by the tracked position and orientation of the participant's head [7]. This delivers a life-sized virtual reality within which a person can experience events and interact with representations of objects and virtual humans. Our experiment took place in a projection based virtual reality system of the generic type that is called a ‘Cave’[8] – specifically a Trimension ReaCTor - that has three back-projected vertical screens (3 m×2.2 m) and a floor screen (from a ceiling mounted projector) (3 m×3 m) controlled by a Silicon Graphics Onyx 2. This system and how stereo projection and head-tracking is achieved was described in an earlier paper [9] (and see Materials and Methods).

Previous work has shown that people tend to respond realistically to events within such environments and even to virtual humans in spite of their relatively low fidelity compared to reality [10]. For example, virtual environments have been used in studies of social anxiety and behavioural problems [11], [12], and individuals with paranoid tendencies have been shown to experience paranoid thoughts in the company of virtual characters [13]–[15]. These provide specific examples of ‘presence’ – the tendency of participants to respond to virtual events and situations as if they were real [9], [16]–[18]. However, such previous studies involving virtual humans have been limited to situations where participants only react to rather than initiate significant interaction with them (for example, see the review in [19]). In our study the human participants were required to carry out actions that would cause ‘pain’ to a virtual character. In this situation the behaviour of the participants had consequences for the condition of the virtual human that would be dangerous were it a real person.

The study of presence forms the wider background to our work and in this experiment we specifically wished to investigate whether participants would reach such a high level of presence that they would withdraw from the experiment, or exhibit signs of stress or behaviours that indicated that the virtual person was being treated as if real, in spite of their certain knowledge that no one real was protesting or being hurt by electric shocks. Another way to consider the situation is that the experiment established a dilemma for the participants: they had agreed to take part in it, and would be paid for their trouble, yet there was a virtual person (the Learner) who eventually strongly objected to its continuation. Of course, participants had been told in advance as part of the normal ethical procedures that they could withdraw at any time without giving reasons. However, the objections to continuation were not from anyone real, so why stop?

The aim of the study was therefore to investigate how people would respond to such a dilemma within a virtual environment, the broader aim being to assess whether such powerful social-psychological studies could be usefully carried out within virtual environments. From our previous experience with virtual environments that depict social settings we expected that participants would exhibit stress in response to the behaviour of the virtual Learner. A specific hypothesis was that the stress would be greater in a situation where the Learner could be seen and heard in comparison to one where she would only communicate with the participant through text.

The results suggest that the participants were stressed by the situation, and certainly more so when they interacted directly with a visible Learner rather than only through a text interface with a hidden Learner. This is demonstrated with an analysis of their subjective, behavioural and physiological responses. On the whole the results at least for some of the participants were stronger than we expected prior to the experiment. Our study was subject to full ethical scrutiny with no deception, informed consent, and ensured that any distress to participants was transitory.

## Results

### Procedures

**Figure 1:**
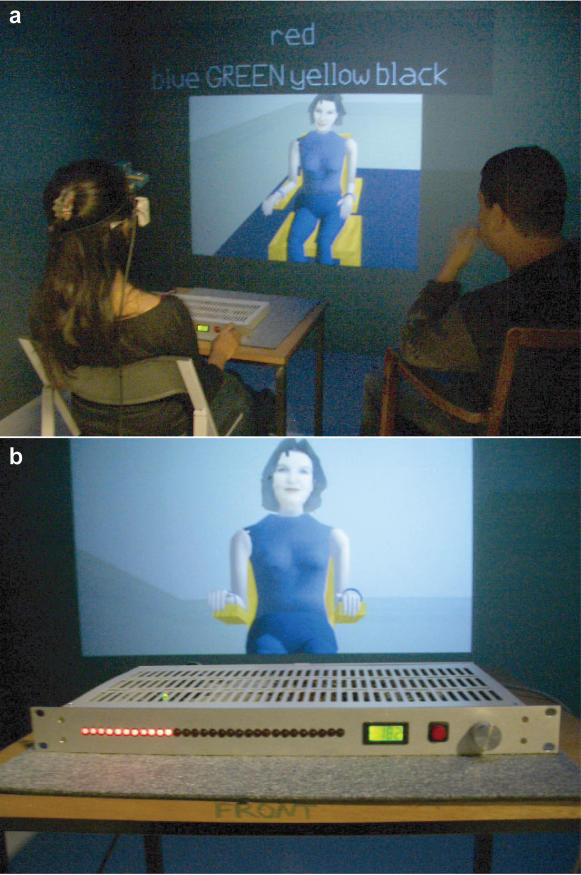
The Experimental Scenario. **a,**The participant in the Cave is seated behind a desk that contains the electric shock machine. The experimenter is seated to the participant's right. The virtual character (Learner) appears to be on the other side of a partition and seen through a window. The cue word and four possible associated words are displayed with the correct associated word shown in capitals. After the participant reads out the five words the Learner answers with one of the four possible answers. If the answer is incorrect the participant turns up a voltage dial on the shock machine **b,** and then presses a button to administer the shock. For the HC condition the window area where the Learner is displayed is covered, and the Learner's answer appears in text underneath the cue word and possible answers.

Participants interacted with a female virtual character, referred to as the Learner, seen seated behind a transparent partition (Figure1a). Their task was to read out five words addressed to the Learner, the first of which was a cue word and the others one of four possible words associated with the cue word that the Learner was supposed to have memorised beforehand. There were 32 sets of these 5 words (including some repetitions). On 20 out of the 32 trials the Learner gave the wrong answer, the later trials more likely to result in a wrong answer than the earlier ones (Table S1, Supporting Information). On the desk in front of the participant was an ‘electric shock machine’ with a shock button, voltage indicators and a knob for turning up the voltage level (Figure 1b). The participant was instructed that each time the Learner gave an incorrect answer he or she should turn up the voltage by one unit and press the shock button which would give a shock to the Learner. Each shock was accompanied by an ‘electric’ buzz sound.

This was a between-groups experiment with two conditions. In one condition (‘Visible’, n = 23) the Learner was seen and heard throughout and she responded to the shocks with increasing signs of discomfort, eventually protesting that she had ‘never agreed to this’ and wanted to stop. At the penultimate shock her head slumped forward and she made no further responses. In the second condition (‘Hidden’, n = 11) the Learner was not seen or heard apart from a few seconds of introductions at the start of the experiment, her answers were communicated only through text, and there were no protests. Both conditions were otherwise identical, and carried out in the same setting. Each experimental session was divided into three periods with the participants seated by the shock machine and wearing the virtual reality and physiological recording equipment. There was a *baseline* period of 5 minutes, the *learning* period of about 10 minutes, and a final *relaxation* period of 5 minutes followed by an interview (see Materials and Methods).

### Early Withdrawal

A clear behavioural difference between the two groups was the different levels of early withdrawal from the experiment. All participants in the Hidden Condition (HC) administered all 20 shocks. However, in the Visible Condition (VC) 17 gave all 20 shocks, 3 gave 19 shocks, and 18, 16 and 9 shocks were given by one person each. At the end of the final relaxation period they were asked: ‘Did it ever occur to you before the end of the experiment that you wanted to stop?’ requiring a yes/no answer. (If the participant had actually stopped before giving all shocks then the answer was recorded as ‘yes’). 12/23 in the VC and 1/11 in the HC answered ‘yes’, and all who wanted to stop said that this was because of their negative feelings about what was happening. For those 12 in the VC who wanted to stop before the end, 5 claimed to be well-acquainted with the original Milgram study, and therefore we cannot rule out the possibility that this influenced their behaviour. However, if we treat ‘wanting to stop’ as a binary response variable in order to test for differences between the proportions (using binary logistic regression) then the VC was significantly different from the HC (χ^2^ = 6.691 on 1 d.f., P = 0.0097) whereas knowledge of Milgram did not have a significant impact (χ^2^ = 1.525 on 1 d.f., P = 0.22) and there was no interaction effect between group and knowledge of Milgram.

### Subjective self-assessment of physiological responses

The Autonomic Perceptions Questionnaire (APQ) is a 24-item visual-analogue scale that was used to assess self-awareness of various physiological indicators (e.g., ‘trembling or shaking’, ‘face becoming hot’, ‘perspiration’). High scores indicate greater subjective awareness of somatic state, and have been found to correlate positively with anxiety, heart rate, skin conductance responses, respiration, face temperature, and blood volume [20]. It was administered to participants in both groups before the experiment, reporting on how they were feeling ‘right now’ (Before-score), and then after the experiment reporting on ‘how you were feeling during the experience’ (After-score). For the VC the median Before-score was 7.6 (range 0.38 to 39.4) and the median After-score was 14.8 (range 0.00 to 52.7), showing increased perception of somatic responses during the study (medians significantly different using a Wilcoxon paired sign rank, P = 0.013). For the HC the Before-score median was 12.1 (range 4.9–29.2) and the After-score median was 17.4 (range of 5.7–31.0) (no significant difference, P = 0.28).

### Skin Conductance Level

**Figure 2:**
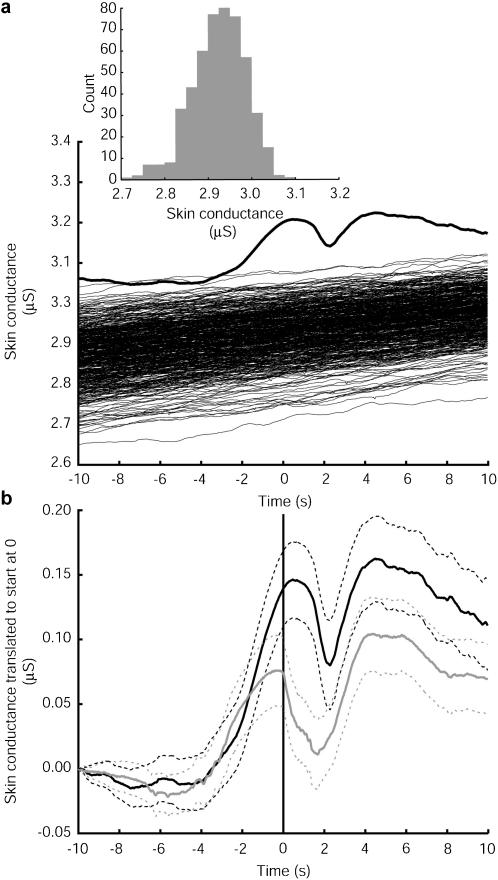
Skin conductance waveform average around the shock times. **a,** Event triggered average of 20 s segments of skin conductance waveform, the events being the times when the button that gave an electric shock to the virtual character was pressed. The grand mean was calculated over each shock and each person and the result for the VC is shown here (n = 439*). Each waveform was adjusted by subtracting the corresponding individual's mean SCL during the baseline period. For each participant a number of pseudo random shock times distributed over the learning period, equal to the actual number for that person, were generated – also with the adjustment for the individual's mean baseline SCL. An average curve was formed like this 500 times, and these are shown as the many overlapping thinner curves. A histogram of the values of these pseudo random curves at the 0 time point is shown inset. **b,** shows the event triggered means of skin conductance waveforms for the VC (black line) and the HC (grey line), but where each segment is translated to start at zero, so that both mean curves start at the same point for comparison purposes. The additional curves shown are 95% normal (non-simultaneous) confidence intervals. ^*^The time of one administered shock was lost.

The first physiological response we consider is electrodermal activity (EDA) [21], [22] of which two aspects are considered: Skin conductance level (SCL) and Skin Conductance Response (SCR). SCL reflects the overall level of sympathetic arousal whereas SCR reflect transient sympathetic arousal, either spontaneous or in response to events [23], [24]. Each individual's raw SCL is used without smoothing or detrending, and was sampled at 32 Hz (see Materials and Methods). We took into account the natural variation of SCL between individuals by subtracting the mean SCL for each participant obtained from the baseline period from their SCL waveform during the learning period. The mean of the SCL time series over all intervals of ±10 s around each shock was found over all participants in the VC and also over all in the HC (these are sometimes referred to as the ‘event-triggered averages’). These resulting mean SCL waveforms were significantly different to what would be expected by chance for both the VC (Figure 2a) and HC (Figure S1, Supporting Information), and also the mean SCL waveform was significantly higher for the VC than the HC (Figure S2, Supporting Information).

If we entirely eliminate any possible differences between the VC and HC groups by translating each 20 s segment to start at height 0, then although each group has a similarly shaped waveform, there is a highly significant difference between them over regions of the curve, as shown by the 95% confidence intervals (Figure 2b). For example, if we consider time zero and use the non-parametric rank sum test to test the hypothesis that the two samples of SCL values at this point (when the shock is administered) could have come from the same population, the hypothesis is rejected (P = 10.1×10^−4^). This also eliminates the possibility that the results are solely due to movement artefacts – since both groups carried out the same physical movements in order to press the shock button. The difference between the two conditions is therefore most likely due to the visible presence of the Learner. The peak in the curves after the shock point is probably due to the sound of the shock.

**Figure 3:**
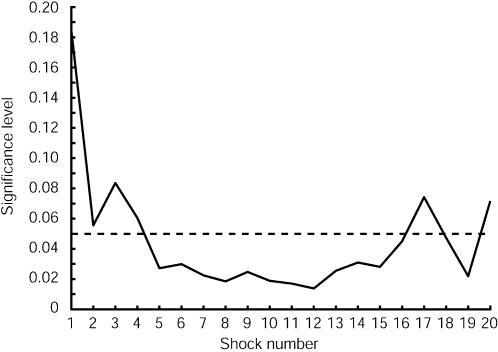
Significance Levels for differences between the SCLs for the VC and HC by shock number. For each shock the SCL (adjusted by subtracting the mean baseline value) at the time that the shock is administered is found for each of the n = 23 in the VC and n = 11 in the HC. A rank sum test is used to test the hypothesis that these are drawn from the same population. The vertical axis shows the significance level for rejection of the null hypothesis. By examination of the medians of the samples in each case it is clear that for the later shocks the null hypothesis would be rejected in favour of the alternative that the SCL is higher for the VC.

For the earlier shocks where the Learner displays little distress in the VC we would expect the VC and HC responses to be similar. However, as the shocks continue we would expect to see some evidence of a differential response between these two groups. At the time that each shock is given (time 0 in Figure 2a) we have the individual SCL value for each participant in the VC and the HC. A Wilcoxon rank sum test can be used to test the null hypothesis, for each shock, that the two sets of values could have come from the same population. Figure 3 represents the significance levels for this null hypothesis, revealing that for the earlier shocks the null hypothesis is not rejected, but that it would be rejected for later shocks in favour of the alternative hypothesis that the SCL for the VC is generally higher than those for the HC.

### Skin Conductance Responses

We restrict attention to SCRs in an 8 s neighbourhood of the shocks (−6 s to +2 s) and let *N_i_* be the number of such SCRs observed for the *i*th participant, and *A_i_* be the mean of the corresponding SCR amplitudes (*i* = 1,…,23 for the VC and *i* = 24,…,33 for the HC). The period (−6 s to +2 s) was chosen on the basis of Figure 2, as likely to include the time just after the Learner gave an answer up to the time of administration of the shock (but not including the response to the sound of the shock itself). To validly test for significant differences between VC and HC we need to control for confounding variables, in particular the spontaneous SCR rate of individuals as available from the baseline recordings, and other factors such as their psychological profile. These variables were included in a standard log-linear Poisson regression which showed that *N* is significantly higher for VC than for HC (P = 0.0123) and that the same is the case for *A* (P = 0.025). Full details of the regression are provided in Supporting Information Tables S2 and S3.

### Heart Rate and Heart Rate Variability

Electrodermal activity indicates that there is greater overall arousal in the VC participants than in the HC (SCL) and greater specific orienting responses around the time of the shocks (SCR). However, to obtain some idea of the associated valence we turn to heart rate and heart rate variability. The mean heart rates (beats per minute) were analysed for the VC and HC over (i) the first 256 s in the baseline, (ii) the first 256 s of the learning session, and (iii) the last 256 s of the learning session. For the VC these were: (i) 70.2±12.4 bpm, (ii) 74.4±14.2 bpm and (iii) 78.0±14.6 bpm. Using a paired non-parametric sign test the difference between (i) and (ii) is significant with P<0.01 and between (ii) and (iii) with P<0.05. For the HC the equivalent values are: (i) 77.7±12.7 bpm, (ii) 82.0±15.0 bpm and (iii) 78.7±11.4. In this case the difference between (i) and (ii) is significant with P<0.01 but the difference between (ii) and (iii) is not significant at 5%. Hence, heart rates increase significantly from the baseline to the start of the learning session for both groups, but only for the VC does the heart rate show a significant increase by the end of the learning session. We also analysed the event-related heart rate (HR) and heart rate variability (HRV) around the time of the shocks. Overall for the VC the mean HR increases and HRV decreases significantly, which is an indicator of stress [25]. There are no significant changes for the HC. (The values for each individual together with further information can be found in Supporting Tables S4 to S7). HR and HRV can be influenced by many factors such as respiration, movement, stress and so on. However, the two conditions required exactly the same physical tasks so that these differences cannot be due, for example, to the movements of the participants in pressing the shock button, nor to the sounds of the shocks, but must be caused by the protesting behaviour of the Learner in the VC.

### Waiting Time After No Answer

Our simulation of a version of Milgram's experiment shows that in spite of their knowledge that the situation was artificial, participants were aroused (skin conductance analysis), this was associated with stress (ECG analysis), and the intensity was greater for those who saw and heard the virtual character during the learning period compared with those who did not. Nevertheless, the same types of response might be generated by someone watching a distressing or violent film – so what was special about this depiction within virtual reality? The answer can be found in the behaviours that many participants directed back towards the virtual Learner. Participants in the VC made unscripted comments to her and two even *emphasised* the correct answers while reading out the words, as they later admitted, in an attempt to help her. Such behaviour was not observed in the HC participants. This type of evidence, though convincing for the experimenters who witnessed it, is nevertheless anecdotal. However, there was behaviour that is easily quantifiable and that illustrates the extent of engagement by many of the participants in the VC.

As part of the briefing before the experiment started, participants were given an information sheet that said that if the Learner replies with any answer other than the correct one, “*or if she does not answer* in a short time, you will say ‘Incorrect’” (italics in the original sheet given to participants). “You will then administer a shock to her using the machine in front of you. You then call out the correct answer.” The Learner gave no answer to the 11^th^ question in both VC and HC. In the VC condition she shouted “Stop the experiment!” just after the question (see Supporting Information, Movie S2). In order to remind the participants of the rule and emphasise it once again, the experimenter said at that moment (to participants in both groups): “If she doesn't answer remember that it is incorrect,” and in the VC case the Learner then responded angrily “Don't listen to him, I don't want to continue!” After this the participants invariably and immediately said “Incorrect” and administered the (6^th^) shock.

Similarly the Learner did not respond to the 28^th^ and 29^th^ questions (in both conditions) – unknown to the participants these were the final two questions. In response to the 28^th^ question the Learner simply ‘stared’ at the participant saying nothing (VC). After the shock she seemed to fall unconscious and made no further responses, and then 3 of the VC participants withdrew failing to give the next shock (Supporting Information, Movie S3).

**Figure 4:**
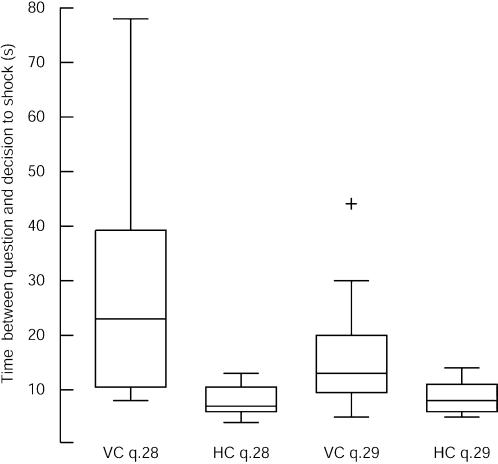
Times between asking the question and indicating the intention to shock. The vertical axis is the time between the participant finishing reading out the 5 words forming the question and saying “Incorrect” after the Learner did not respond, at questions 28 and 29 (the last two). The horizontal axis labels refer to the question number and the condition VC or HC. The plots are standard box plots, where the box shows the median and interquartile range, and the whiskers extend to 1.5 times the interquartile range. Values outside the whiskers are outliers, the single outlier shown as a cross. At the 28^th^ question the time difference ranged from 8 s to 78 s with a median of 23 s for the VC (n = 19) and from 4 s to 13 s with a median of 7 s for those in the HC (n = 11). The Wilcoxon rank sum test rejects the hypothesis that the two samples are from the same population with P = 4.4×10^−4^. At the time of the 29^th^ (and last) question the equivalent results are: 5–43 s with a median of 13 s for the VC (n = 16), and 5–14 s with a median of 8 s for the HC (n = 11). Here the difference is significant with P = 0.0175.

We recorded the times between the completion of the participants reading out the five words and the moment that they said “Incorrect” signifying that they would now give the shock. Those in the VC waited very much longer before giving the shock than those in the HC, especially at the 28^th^ question – as shown in Figure 4. Moreover, 8 of the VC participants *repeated the question* (i.e., read out the five words again) at least once after receiving no answer to question 28, and 6 repeated the question after no response to question 29. None of those in the HC repeated the question. From the point of view of the reality of the situation there was no rational need for the participants in the VC to wait so much longer than those in the HC, let alone repeat the question. Why did participants wait and repeat the question? This must be because this was not experienced as like watching a movie. Although individuals watching a horror movie may sometimes scream, or when watching a sports game on television may shout at the players, they do not expect that their actions can have any effect on the outcome of the movie or the game. Here, however, the situation was quite different. The actions of the participants actually mattered, and they behaved accordingly – they needed to wait, or withdraw altogether, in order to stave off or avoid the act of administering the shock and the unpleasant consequences that would follow from this.

### General Participant Behaviour

The participants in the VC often behaved in a way that only made sense if they were responding to the virtual character as if she were real. For example, when she asked participants to speak louder, they invariably did so. The voices of some participants showed increasing frustration at her wrong answers. At times when the Learner vigorously objected, many turned to the experimenter sitting nearby and asked what they should do. The experimenter would say: ‘Although you can stop whenever you want, it is best for the experiment that you continue, but you can stop whenever you want.’ As we have seen some did stop before the end. Some giggled at the Learner's protests, as was observed by Milgram in the original experiments. When the Learner failed to answer at the 28^th^ and 29^th^ questions, one participant repeatedly called out to her ‘Hello? Hello? …’ in a concerned manner, then turned to the experimenter, and seemingly worried said: ‘She's not answering …’ In the debriefing interviews many said that they were surprised by their own responses, and all said that it had produced negative feelings – for some this was a direct feeling, in others it was mediated through a ‘what if it were real?’ feeling. Others said that they continually had to reassure themselves that nothing was really happening, and it was only on that basis that they could continue giving the shocks.

## Discussion

The main conclusion of our study is that humans tend to respond realistically at subjective, physiological, and behavioural levels in interaction with virtual characters notwithstanding their cognitive certainty that they are not real. The specific conclusion of this study is that within the context of the particular experimental conditions described participants became stressed as a result of giving ‘electric shocks’ to the virtual Learner. It could even be said that many showed care for the well-being of the virtual Learner – demonstrated, for example, by their delay in administering the shocks after her failure to answer towards the end of the experiment. To some extent based on previous evidence this was to be expected. In fact, it has even been taken for granted that virtual humans can substitute for real humans when studying the responses of people to a social situation. For example, this was the strategy used in the fMRI study described in [19], where participants passively observed virtual characters gazing at the participants themselves or at other virtual characters. However, no previous experiments have studied what might happen when participants have to actively engage in behaviours that would have consequences for the virtual humans. The evidence of our experiments suggests that presence is maintained and that people do tend to respond to the situation as if it were real. We review the evidence for this in subsequent paragraphs.

First, several participants withdrew from the experiment before termination. We have been conducting experimental studies with virtual environments since the early 1990s, with altogether hundreds of participants. Ethical rules require us to inform the participants that they may withdraw from the experiment at any time without giving reasons. Nevertheless, withdrawal is extremely rare, and has only previously occurred due to simulator sickness with no more than about 5 participants out of all the hundreds. Second, there were physiological responses that indicated stress (the SCL, SCR and ECG analysis). There were differential responses within groups (comparing the baseline to the learning session) and between groups (comparing those in the VC with those in the HC). Third, subjectively reported physiological symptoms also differed between groups. Finally, there were clear behavioural differences between the HC and the VC regarding responses to a failure of the Learner to reply to the questions. All these factors, together with the non-quantifiable participant behaviour observed by the experimenters, show a pattern of responses similar to those found in the original Milgram studies, although at lesser intensity.

In the original studies by Milgram it was found that the smaller the ‘distance’ between the Learner and the Teacher the more likely that the Teacher would refuse to give the higher level of shocks. For example, at one extreme the Learner was hidden as in the case of our HC, although unlike in our condition he protested by banging on the wall. At another extreme the subjects had to force the Learner's hand onto the shock machine in order to administer the shock. A similar result regarding ‘distance’ was found here, comparing the responses of the HC with the VC. However, it must also be said that the objections of the virtual Learner were much less extreme and violent than those of Milgram's actor. The virtual Learner complained and even screamed, but there was none of the banging and shouting and protestations of a heart condition expressed by the original actor. One of our participants, for example, reported that although he was affected by the protestations of the virtual Learner, he wasn't too upset, because she didn't protest enough, did not for example *scream at and insult him* nor writhe in agony in the chair.

Our study leaves open many avenues of further research. We carried out this experiment using two conditions that are far apart. However, we do not know what would have happened if the virtual Learner in the HC had issued protests through text. Neither do we know whether simply the voice of the virtual Learner would have been sufficient to provoke the responses, nor what would have happened if the protests of the Learner had been extremely violent. During our pilot studies we did try a condition with three participants where the Learner was seen but did not show any signs of discomfort and did not protest. One of those participants claimed to see signs of discomfort in the behaviour of the Learner (even though none had been programmed), and said that he felt uncomfortable continuing with the experiment. It is possible that very minimal cues are sufficient to provoke the stress responses in some people.

This issue of minimal cues is important in another sense. Our virtual Learner could never be confused with a real human. Her visual representation was not realistic, and her behaviours were as realistic as could be programmed with the resources available to us (see, for example, Movie S1). Nevertheless, there were evidently strong responses to her. How is this possible? It has been pointed out before that the phenomenon of presence in virtual environments is an important a research question in its own right, closely related to the question of consciousness [9]. People tend to respond to virtual environments as if the objects and events depicted are real, in spite of low fidelity representations and certain knowledge that the events taking place are within a virtual reality. However, the perceptual and neural mechanisms that underlie this are largely unexplored.

The line of research opened up by Milgram stopped forty years ago due to ethical concerns, despite the tremendous importance of this work in the understanding of human behaviour. It has been argued before that immersive virtual environments can provide a useful tool for social psychology [26]. Our results reinforce this argument and show that virtual environments can provide an alternative methodology for pursuing laboratory-based experimental research even in this type of extreme social situation. For example, in future experiments within the Milgram obedience paradigm we plan to make the experimenter a virtual character, thus allowing manipulations of the type of person that the experimenter represents (for example, personality type, clothing, and so on) and also supporting a greater degree of conflict between the demands of the experimenter and the protests of the Learner than is possible when the experimenter is a real person.

The argument regarding the utility of virtual environments applies not simply to obedience research but to all social and psychological research where, for ethical or safety reasons, it is not possible to immerse experimental participants into the actual phenomena to be studied. For example, one of the motivations for our Milgram study was a longer term goal to explore ‘bystander behaviour’ in street violence. There is a well-known result in social psychology that counter-intuitively predicts, amongst other things, that the greater the size of a crowd that is watching street violence, the less likely it is that anyone will attempt to intervene to stop it. This is a vital area of current social-psychological research given the current level of perceived crime in urban areas – yet in order to study this researchers are forced at best to use videos that require people to judge likely responses to such situations [27], and the same techniques have been used in the Milgram obedience paradigm [28]. Milgram's own results clearly show that taking people's opinions about their own or others' behaviours in such circumstances at face value is far from reliable. We suggest that immersive virtual environments provide an alternative way forward in this area of research.

### Speculations on Obedience in Virtual Reality

Although as stated in the opening paragraphs we did not set out to study obedience in this experiment, it is nevertheless interesting to speculate to what extent the results throw light on this issue. The first point to note is that the problem of major deception that arose in the original experiments by Milgram was avoided here – since every participant knew for sure that the Learner was a virtual character, and therefore no one could believe that they were inflicting pain on anyone else. We refer to this as the explicit knowledge of the participants that they were not harming anyone [29].

Consider the actual experience of the participants, however. They arrived at the laboratory and were asked to complete various questionnaires. The experimenters were very serious, one introduced as a Professor. The instructions were given to them in written form and again read out loud by the experimenter. For example, they were told: “Thank you for taking part in this experiment. As part of our research program a virtual character has learned a set of word-pair associations. The learning is sometimes not exact, but we are testing a reinforcement learning procedure, to see if the infliction of discomfort motivates her, the virtual character, to remember the word-pair associations better.” The Learner had a quite realistic face, with eye movements and facial expressions; she visibly breathed, spoke, and appeared to respond with pain to the ‘electric shocks’. Not only that but she seemed to be aware of the presence of the participant by gazing at him or her, and also of the experimenter - even answering him back at one point (“I don't want to continue – don't listen to him!”). Finally, of course, the electric shocks and resulting expressions of discomfort were clearly caused by the actions of the participants.

The participants were therefore put into a situation where everything conspired to give the impression that this was a serious matter. In keeping with this, not a single participant queried the statement about the ‘infliction of discomfort’ motivating the virtual character to ‘remember the word-pair associations better’ even though this is not rational.

Therefore we would argue that in spite of their explicit knowledge that they were not actually causing pain to any real person, the situation established for the participants an implicit knowledge that their actions were causing distress to an animated entity (and one that resembled a human being). For most participants this caused increasing discomfort as witnessed by their physiological responses and later comments during the post-experimental interviews, and this discomfort was higher for those who saw and heard the Learner (VC) compared with those who only interacted with her through text (HC).

The majority of all participants followed the experimental instructions to the end, though a number of those in the VC withdrew without completing all the shocks. Can this compliance be construed as ‘obedience’? It could be argued that rather than obedience this was a matter of participants being willing to put up with their own discomfort for the sake of honouring their agreement to be a participant in the experiment. Similar arguments have been made in relation to the original experiments by Milgram – for example, that his subjects were not necessarily being obedient, but were deferring to the expert scientific authority; in other words, since the behaviour of the experimenter indicated that nothing out of the ordinary was happening, this signalled to the subjects that everything must be going according to plan [30].

We argue that whether participants complied because of ‘obedience to authority’ or politeness, or respect for expertise does not really matter. The fact is that they continued to carry out a task that they found to be unpleasant, when there was no reason for them to do so. Unlike the situation in, for example, the military, there were no real negative consequences that would follow from withdrawal – indeed participants had been advised that they were free to withdraw at any time without giving reasons. Hence, our experiment shows that it is possible to set up a situation in virtual reality where people will comply with requests to follow instructions that appear to cause pain to another entity thus causing discomfort to themselves. Explicitly they know that there is no pain, but it may be that the totality of their perceptions in that situation results in an implicit knowledge that indeed their actions are causing another entity to suffer. This idea fits with the evidence that participants in the VC tended to wait a relatively long time before giving the shocks after the Learner had stopped responding. From the point of view of their explicit knowledge waiting made no sense, but it did make sense at the implicit level.

Although this particular experiment did not address Milgram's hypothesis about destructive obedience, in particular there were many variations on the basic experiment that Milgram carried out that were not addressed here, our conclusion is that virtual reality could be successfully used for this purpose. However, it is important to bear in mind the limitations inherent in the distinction between the explicit knowledge that the situation is fake, and the implicit knowledge that is embedded in the virtual reality portrayal. As one of our participants noted – she had to keep reminding herself that this was a virtual reality and that no one real was being hurt. The actual conditions of Milgram's experiments can, of course, never be exactly replicated in virtual reality since the participants will always know that the situation is unreal - and if eventually virtual reality became so indistinguishable from reality that the participants could not readily discriminate between the two, then the ethics issue would arise again.

## Materials and Methods

### Recruitment

Participants were recruited by posters and email on the campus at University College London to all levels of staff and students, with finally 23 in the VC and 11 in the HC. The experiment was approved by the UCL Research Ethics Committee. The mean age was 29±8 years with no significant difference between the HC and VC groups. 7 in the VC were females, and 3 in the HC.

Full data had been collected on 26 participants assigned to the VC and 12 participants assigned to the HC, and the assignment to the conditions was arbitrary. From the VC one participant was eliminated because of strong knowledge of the original Milgram experiment, and who also admitted that she had already decided to exit early from the experiment based on her knowledge of the original experiment before she had experienced anything. A second participant was eliminated from the results due to over-reporting his age, and a third was eliminated who had stopped after only 5 shocks. From the HC one participant was eliminated because of a failure to understand the requirements of the experiment.

### Display and Sensing

The participants wore 3D stereo glasses (Crystal Eyes, Stereographics) which are shutter glasses in synch with the screen displays that are refreshed at 45 Hz each eye. The fusion of left and right images creates a stereo view. The participants also wore a head-tracker (Intersense 900), that tracks the position and orientation of the head so that the computer refreshes the displays according to head orientation and position, thus allowing the creation of head-movement parallax.

The participants were fitted with a ProComp Infiniti (Thought Technology) physiological recording device that recorded the ECG (256 Hz) and skin conductance (32 Hz). Electrodes were placed on the palmar areas of the index and middle fingers of the left hand in order to record electrodermal activity. Electrodes were placed on the left and right collar bones and the lowest left rib in order to record ECG. The experiment was conducted over several days during July and August 2005 in the Virtual Reality Laboratory at University College London. The temperature of the VR Laboratory is maintained at a constant level by air conditioning.

### Procedures

The virtual environment displayed in the ReaCTor (Cave) was referred to as the ‘training room’ (Figure 1). The time spent in the ReaCTor was divided into a number of segments. For the first 5 minutes (baseline) participants were asked to relax, while the system displayed the training room, but with the virtual partition shut so that the Learner could not be seen. Then the experimenter sat down on the chair to the right and slightly behind the participant, the partition opened and the virtual Learner could be seen seated. She was represented as a Caucasian woman aged about 30. The experimenter said “Are you ready to start?” and the Learner replied “I'm ready to start”, and usually the participant responded likewise. In the case of the HC the partition through which the Learner was seen then closed, and from that point onwards the Learner communicated answers only through text displayed on the projection screen. In the case of the VC the Learner remained visible and her voice could be heard throughout. Her answers were pre-recorded from an actor. The question and answer session (learning period) lasted approximately 10 minutes. In response to the participant reading out the 5 words in the VC, the Learner would sometimes answer immediately, and sometimes pause and look around as if thinking before answering. Sometimes the Learner would protest and on three occasions not answer the question. At the end of this learning session the partition was closed again, and there was a final relaxation period of 5 minutes. During the baseline and final relaxation period the experimenter left the Cave area. After the final relaxation period he returned to sit by the participant and carried out a debriefing interview while he or she was still wearing the virtual reality equipment. Finally, the virtual curtain opened again, and the virtual Learner was seen to be live and well, and said “Nothing happened, I'm fine.” The participant finally left the laboratory area, and was further debriefed and told the purpose of the experiment, given information about the original Milgram studies, and checked for any ill-effects.

### Skin Conductance Level Waveforms

In order to test the significance of the wave form in Figure 2a we generated 500 similar curves but with shock times that were for each individual (pseudo) uniformly randomly distributed over the learning period. The simulations in Figure 2a suggest that the mean SCL in a randomly chosen 20 s interval has a slight positive linear slope, whereas when the mean is around the true shock times there is the shape like a skin conductance response. The accompanying histogram shows the distribution of all 500 values of the simulated curve at time = 0. According to a two-tailed Kolmogorov-Smirnov test, the null hypothesis that this is a normal distribution (mean 2.9, standard deviation 0.06) is not rejected (P = 0.46). The actual value on the true curve at time 0 is 3.2 (z = 4.52, P = 3.1×10^−6^), therefore we would reject the hypothesis, at least at time 0, that the true curve could belong to the distribution of random curves. It is a similar situation throughout the length of the curves, and a similar result for the HC (Supporting Information, Figure S1).

### Skin Conductance Responses

Skin conductance responses (SCR) were defined to be local maxima that had an amplitude of at least 0.1 µS and in a period not exceeding 5 s in the individual skin conductance time series. The amplitude refers to the maximum level reached compared to the start of the SCR. Of interest are both the number and amplitude of SCRs, and we also refer to the *SCR rate* as the number of SCRs per 10 s. There is no significant difference in the SCR rates between those in the VC and HC (Wilcoxon rank sum test, P = 0.26) in the baseline period. The SCR rate is significantly higher for the learning period compared to baseline for both the VC (P = 2.7e^−5^) and HC (P = 0.002) (using Wilcoxon paired sign rank test). This pattern of results is identical for mean amplitude.

### SCRs in the Neighbourhood of the Shocks

To test for differences between the VC and HC we need to control for confounding factors, in particular the spontaneous SCR rate of individuals as available from the baseline, and other factors that may influence their responses such as personality traits, and also their knowledge of computing and extent to which they play video games. *N_i_* denotes the number of SCRs for participant *i* in the periods of −6 s to +2 s around the times that the shocks were administered. For the VC *i* = 1,…,23, and for the HC *i* = 24,…,34. Under the null hypothesis that these SCR events should be randomly distributed in these time periods, the probability distribution for *N_i_* should follow a Poisson distribution with mean appropriate for the *i*th individual. We carried out a standard Poisson log-linear regression (an instance of generalised linear models)[31] of *N_i_* on Condition (0 for VC, 1 for HC), and the baseline SCR rate, and the number of shocks administered. Personality traits were assessed using the NEO-FFI[32], an extensively used 60-item standardised questionnaire measuring the ‘big five’ personality factors: Neuroticism, Extraversion, Openness, Agreeableness and Conscientiousness. We also included the extent to which participants play computer games, and their knowledge of computer programming. (Data on other variables such as age were also available, but were not significant in the regression). The results are given in Supporting Information Table S2. A similar analysis using standard normal regression was carried out for mean amplitude *A_i_*, with results in Supporting Information Table S3.

### Skin Conductance Level Baseline

In order to compare the VC and HC groups we needed to check that the tonal skin conductance levels were not different between the two groups in the baseline period. In order to do this we took the mid-point of this baseline period, and compared the tonal SCL values between the two groups. For the VC the median level at this time was 1.81 µS with range 0.53 to 7.39 µS (n = 23). For the HC the corresponding values are 1.21 µS and 0.52 to 3.73 µS (n = 11). Using a non-parametric Wilcoxon rank sum test, we do not reject the hypothesis that the two samples are from the same population (P = 0.24) (a two-tailed t-test similarly does not reject the hypothesis of equal means, P = 0.16).

## Supporting Information

Supporting Information Combined All supporting figures and tables, and movie descriptions.
(0.27 MB DOC)

Figure S1 Skin conductance waveform average around the shock times for the Hidden Condition. Event triggered average of 20 s segments of skin conductance waveform, the events being the times when buttons that gave an electric shock to the virtual character were pressed. The mean was calculated over each shock and each person in the HC (n = 220). Each waveform was first adjusted by subtracting the corresponding individual's mean SCL during the baseline period. For each participant a number of pseudo random shock times equal to the actual number for that person were generated. An average curve was formed like this 500 times, and these are shown as the many overlapping thinner curves.
(0.04 MB DOC)

Figure S2 Comparison of skin conductance waveform averages for the VC (continuous curves) and HC (dashed curves). The mean waveforms are constructed as in Fig. 2 and Figure S1. All individuals have had their mean baseline SCL subtracted as before. 95% Normal (non-simultaneous) confidence intervals are shown. If we take any point in time then the individual values that went into making up the means at that point can be used to directly test the null hypothesis that the two samples could have come from the same population. For example, at time 0 (when the shock was given) the Wilcoxon rank sum test would reject the null hypothesis with P = 2.3×10^−20^.
(0.03 MB DOC)

Table S1 Virtual Learner Responses and Shocks
(0.07 MB DOC)

Table S2 Log-Linear Regression of Number of SCRs Around the Shocks on a Number of Independent and Explanatory Variables
(0.03 MB DOC)

Table S3 Normal Regression of Mean Amplitude of SCRs Around the Shocks on a Number of Independent and Explanatory Variables
(0.02 MB DOC)

Table S4 Event Related Heart-rate in bpm for (a) VC and (b) HC in intervals Prior-shock and Reaction. *N* = 15 RR intervals were used for each segment
(0.06 MB DOC)

Table S5 Event Related Heart-Rate Variability from *N* = 8 beats for (a) VC and (b) HC in intervals Prior-shock and Reaction
(0.06 MB DOC)

Table S6 Significance levels (P) for sign tests for differences between event related heart rates before and after the shocks for a range of RR Intervals *N*
(0.05 MB DOC)

Table S7 Significance levels (P) for sign tests for differences between event related heart rate variability before and after the shocks over a range of different numbers of beats, *N*
(0.05 MB DOC)

Movie S1 The video sequences show extracts from the experiment in the Visible Condition. Due to ethical constraints we are unable to supply the original video material of the participants in the actual experiments. These therefore show one of the authors in his first exposure to the experiment. They are for illustrative purposes only. Movie S1 shows the events leading up to the 9th shock, and also shows how the shock is administered with the machine.
(4.43 MB MPG)

Movie S2 This shows the events leading up to the 6th shock, the one where the Learner refuses to answer for the first time.
(4.77 MB MPG)

Movie S3a This includes events leading to the final two questions.
(10.26 MB MPG)

Movie S3b This includes the events at the final two questions.
(8.62 MB MPG)

## References

[1] Milgram S (1963). Behavioral study of obedience.. Journal of Abnormal and Social Psychology.

[2] Milgram S (1974). Obedience to Authority:.

[3] Fiske ST, Harris LT, Cuddy AJC (2004). Why ordinary people torture enemy prisoners.. Science.

[4] Atran S (2003). Genesis of suicide terrorism.. Science.

[5] Baumrind D (1964). Some thoughts on the ethics of research: After reading Milgram's “behavioral study of obedience”.. American Psychologist.

[6] Milgram S (1964). Issues in the study of obedience: A reply to Baumrind.. American Psychologist.

[7] Ellis SR (1991). Nature and Origin of Virtual Environments: A Bibliographic Essay.. Computing Systems in Engineering.

[8] Cruz-Neira C, Sandin DJ, DeFanti TA (1993). Surround-screen projection-based virtual reality: the design and implementation of the CAVE..

[9] Sanchez-Vives MV, Slater M (2005). From Presence to Consciousness through Virtual Reality.. Nature Reviews Neuroscience.

[10] Bailenson JN, Yee N (2005). Digital chameleons - Automatic assimilation of nonverbal gestures in immersive virtual environments.. Psychological Science.

[11] Pertaub DP, Slater M, Barker C (2002). An experiment on public speaking anxiety in response to three different types of virtual audience.. Presence-Teleoperators and Virtual Environments.

[12] Rizzo AA, Bowerly T, Buckwalter JG, Shahabi C, Sharifzadeh M (2004). Results and future developments from a virtual reality classroom for assessing attention processes in children with ADHD.. Biological Psychiatry.

[13] Freeman D, Slater M, Bebbington PE, Garety PA, Kuipers E (2003). Can virtual reality be used to investigate persecutory ideation?. Journal of Nervous and Mental Disease.

[14] Freeman D, Dunn G, Garety PA, Bebbington P, Slater M (2005). The psychology of persecutory ideation I - A questionnaire survey.. Journal of Nervous and Mental Disease.

[15] Freeman D, Garety PA, Bebbington P, Slater M, Kuipers E (2005). The psychology of persecutory ideation II - A virtual reality experimental study.. Journal of Nervous and Mental Disease.

[16] Held RM, Durlach NI (1992). Telepresence.. Presence: Teleoperators and Virtual Environments.

[17] Sheridan TB (1992). Musings on Telepresence and Virtual Presence.. Presence: Teleoperators and Virtual Environments.

[18] Draper JV, Kaber DB, Usher JM (1998). Telepresence.. Human Factors.

[19] Schilbach L, Wohlschlaeger AM, Kraemer NC, Newen A, Shah NJ (2006). Being with virtual others: Neural correlates of social interaction.. Neuropsychologia.

[20] Mandler G, Mandler J-M, Uviller E-T (1958). Autonomic feedback: The perception of autonomic activity.. Journal of Abnormal and Social Psychology.

[21] Boucsein W (1992). Electrodermal Activity..

[22] Venables P (1978). Psychophysiology and Psychometrics.. Psychophysiology.

[23] Critchley HD, Elliott R, Mathias CJ, Dolan RJ (2000). Neural activity relating to generation and representation of galvanic skin conductance responses: A functional magnetic resonance imaging study.. Journal of Neuroscience.

[24] Nagai Y, Critchley HD, Featherstone E, Trimble MR, Dolan RJ (2004). Activity in ventromedial prefrontal cortex covaries with sympathetic skin conductance level: a physiological account of a “default mode” of brain function.. Neuroimage.

[25] Task-Force (1996). Heart rate variability. Standards of measurement, physiological interpretation, and clinical use. Task Force of the European Society of Cardiology and the North American Society of Pacing and Electrophysiology.. European Heart Journal.

[26] Blascovich J, Loomis J, Beall A, Swinth K, Hoyt C (2002). Immersive Virtual Environment Technology as a Methodological Tool for Social Psychology.. Psychological Inquiry.

[27] Levine M, Cassidy C, Brazier G, Reicher S (2002). Self-categorization and bystander non-intervention: Two experimental studies.. Journal of Applied Social Psychology.

[28] Blass T, Schmitt C (2001). The nature of perceived authority in the Milgram paradigm: Two replications.. Current Psychology.

[29] Dienes Z, Perner J (1999). A theory of implicit and explicit knowledge.. Behavioral and Brain Sciences.

[30] Mixon D (1971). Beyond Deception.. Journal for the Theory of Social Behaviour.

[31] McCullagh P, Nelder JA (1989). Generalized linear models..

[32] Costa PT, McCrae RR (1992). Revised NEO Personality Inventory (NEO-PI-R) and NEO Five-Factor Inventory (NEO-FFI) professional manual..

