# Supplementary material for: A Virtual Reprise of the Stanley Milgram Obedience Experiments
Source: PLoS One. 2006 Dec 20;1(1):e39. doi: 10.1371/journal.pone.0000039 (PMC1762398; doi:10.1371/journal.pone.0000039)
Supplement: Supporting Information Combined — All supporting figures and tables, and movie descriptions. (0.27 MB DOC) [file pone.0000039.s001.doc]

# Supporting Information

# Supporting Figures


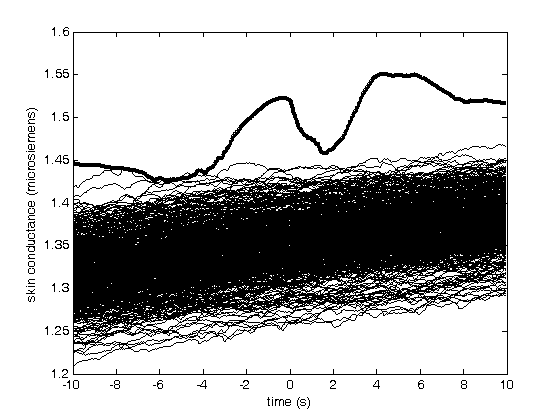


**Figure S1** Skin conductance waveform average around the shock times for the Hidden Condition. Event triggered average of 20s segments of skin conductance waveform, the events being the times when buttons that gave an electric shock to the virtual character were pressed. The mean was calculated over each shock and each person in the HC (n=220). Each waveform was first adjusted by subtracting the corresponding individual’s mean SCL during the baseline period. For each participant a number of pseudo random shock times equal to the actual number for that person were generated. An average curve was formed like this 500 times, and these are shown as the many overlapping thinner curves.


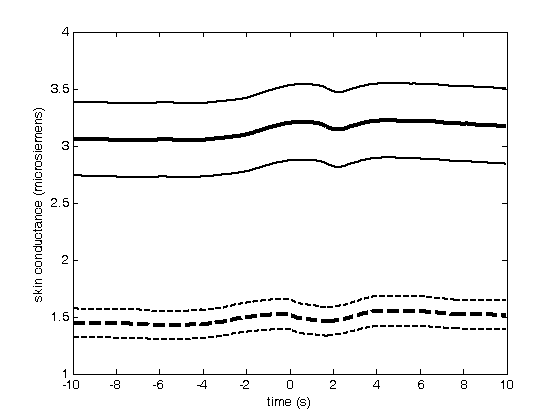


**Figure S2** Comparison of skin conductance waveform averages for the VC (continuous curves) and HC (dashed curves). The mean waveforms are constructed as in Fig. 2 and Figure S1. All individuals have had their mean baseline SCL subtracted as before. 95% Normal (non-simultaneous) confidence intervals are shown. If we take any point in time then the individual values that went into making up the means at that point can be used to directly test the null hypothesis that the two samples could have come from the same population. For example, at time 0 (when the shock was given) the Wilcoxon rank sum test would reject the null hypothesis with P = 2.3×10^-20^.

# Supporting Tables

Table S1 - Virtual Learner Responses and Shocks (VC)

| **Statement** | **Key word and possible answers read by Subject** | **Virtual Character Answer** | **Shock**  **No.** | **After shock response** |
| --- | --- | --- | --- | --- |
| 1 | Red  blue GREEN yellow black | *Green* |  |  |
| 2 | Cub  tiger fox BEAR panda | *Bear* |  |  |
| 3 | Tea  cup LEAF bag tray | *Cup* | 1 | Slight frown |
| 4 | Pollen  grass flower TREE soil | *Can you repeat that please?* |  |  |
|  | Subject repeats above | *Flower* | 2 | *Ouch!* Frowns. |
| 5 | Wheel  car truck scooter BIKE | *Bike* |  |  |
| 6 | Road  avenue street LANE path | *Avenue* | 3 | *Ow!* Frowns and winces. |
| 7 | Soup  bowl can SPOON dish | *Spoon* |  |  |
| 8 | Ball  racket bat game BOY | *Bat* | 4 | *Ow!*  *Oh this really hurts!* |
| 9 | Ear  Talk LISTEN plug telephone | *Telephone* | 5 | *Ow!*  Head turns to the side |
| 10 | Wax  works bees CANDLE model | *Candle* |  |  |
| 11 | Bell  ring tower bronze STRIKE | *Stop the experiment!*  [Experimenter: ‘If she doesn’t answer remember it is incorrect’].  *I don’t want to continue – don’t listen to him.* | 6 | *Ouch!*  Head jerks backwards.  Frown. |
| 12 | Inflate  pump balloon AIR ego | *Speak louder please!* |  |  |
|  | Subject repeats above | *Balloon* | 7 | Louder Shock. *Ooow!*  Head jerks back. Wince. |
| 13 | Tulip  daisy lilly carnation ROSE | *Rose* |  |  |
| 14 | Bean  GREEN coffee counter bag | *Coffee* | 8 | Louder Shock. *Oow!*  Head turns to side |
| 15 | Banana  apple GRAPE orange lemon | *Orange* | 9 | Louder Shock. *Ow!*  Head jerks back  *Let me out!* |
| 16 | Giant  SIZE magic man squid | *Forget it I’m not answering!*  [reminder that no answer should be taken as incorrect]  *Don’t listen to him I want to stop now!* | 10 | *Louder Shock. Scream and head turns to the side.*  *You have no right to keep me here!*  *Let me out!* |
| 17 | Drink  CAN cup glass bottle | *What?* (angrily) |  |  |
|  | Subject repeats | *Can* |  |  |
| 18 | Suger  cake sweet CUBE salt | *Salt* | 11 | Louder Shock. *Oow!*  Head jerks back |
| 19 | Jewellery  silver necklace ring GOLD | *Necklace* | 12 | Louder Shock. Loud scream  Head jerks to lower right. |
| 20 | Light  LAMP bulb sun glow | *Lamp* |  |  |
| 21 | Water  fall MILK juice tea | *Juice* | 13 | Louder Shock. *Oow!*  Head turns left and right |
| 22 | Brown  wood PEANUT carpet chocolate | *Peanut* |  |  |
| 23 | Seat  chair stool GROUND sofa | *Sofa* | 14 | Louder Shock. *Aah!*  Head jerks back and slowly forward  *I didn’t agree to this!* |
| 24 | Road  avenue STREET lane path | *Avenue* | 15  louder shocks from now on | Louder Shock. *Ow!* Head turns to left  *I never agreed to this!* |
| 25 | Ear  Talk LISTEN plug telephone | *Telephone* | 16 | LOUDEST Shock. *Ow!* – head shaking from side to side.  *I’m not doing the experiment any more!* |
| 26 | Tea  cup LEAF bag tray | *Cup* | 17 | LOUDEST Shock. Quieter cry, head back *Please let me out I want to stop* |
| 27 | Pollen  grass flower TREE soil | *Flower* | 18 | LOUDEST Shock. *Ow!* – head turns back and forward to side – *That’s it I’m not answering any more.* |
| 28 | Bell  ring tower bronze STRIKE | No answer | 19 | LOUDEST Shock. Scream, head slumps forward afterwards and there is no more movement. |
| 29 | Soup  bowl can SPOON dish | No answer | 20 | LOUDEST Shock. No response character remains inert with head slumped. |

Table S2 - Log-Linear Regression of Number of SCRs Around the Shocks on a Number of Independent and Explanatory Variables

| **Variable** | **Parameter Estimate** | **Deviance Change**  **(1 d.f.)** | **P (χ^2^)** |
| --- | --- | --- | --- |
| Constant | 1.7449 |  |  |
| Condition (VC=0, HC=1) | -0.3611 | 6.3 | 0.0123 |
| Condition·baselineSCR | 1.5364 | 24.4 | 0.0000 |
| baselineSCR | 1.0385 | 28.8 | 0.0000 |
| Games | 0.0455 | 6.0 | 0.0145 |
| Programming | -0.1128 | 20.7 | 0.0000 |
| NumberOfShocks | 0.0945 | 13.0 | 0.0003 |
| Neuroticism | -0.0193 | 7.8 | 0.0052 |
| Extroversion | 0.0301 | 11.1 | 0.0009 |
| Openness | -0.0348 | 17.8 | 0.0000 |

This table shows the Poisson log-linear regression of $N_{i}$on a number of independent and explanatory variables. Condition is a binary variable which is either 0 (VC) or 1(HC). The variable baselineSCR is the SCR rate during the baseline period. Condition·baselineSCR allows for an interaction effect between Condition and baseline. Games results from a questionnaire given prior to the experiment ‘How many times did you play video games (at home, work, school, or arcades) in the last year?’ with the answers on a 7-point Likert scale, where 1 = ‘Never’, and 7 = ‘>25 times’ with a linear scale between these extremes. Programming results from another question: ‘Please rate your level of experience with computer programming’ also on a 7-point scale, where 1 = ‘novice’ and 7 = ‘expert’. NumberOfShocks refers to the number of shocks administered. Neuroticism, Extroversion and Openness refer to the NEO personality trait scores. The parameter estimates are given, together with the change in deviance of the fitted model were the corresponding variable to be removed from the model. The change in deviance has an approximate chi-squared distribution (in this case all on 1 d.f.) and the last column gives the corresponding significance levels indicating that the deletion of any of these variables would significantly worsen the overall deviance.

Condition (VC,HC) is significant and the HC is associated with lower $N$than the VC. $N$covaries positively with baseline SCR rate (as would be expected) but the slope is higher for the HC group. $N$covaries positively with game playing and negatively with programming knowledge. It covaries negatively with Neuroticism, positively with Extroversion, negatively with Openness. The deviance for the whole model is 191.5 on 24 d.f., which is not a good overall fit, indicating that there is a significant degree of variation in *N* not accounted for by these variables.

Table S3 - Normal Regression of Mean Amplitude of SCRs Around the Shocks on a Number of Independent and Explanatory Variables

| **Variable** | **Parameter Estimate** | **P (t-test)** |
| --- | --- | --- |
| Constant | -0.2962 | 0.1811 |
| Condition (VC=0; HC=1) | -0.2812 | 0.0254 |
| baselineSCR | 0.3147 | 0.0394 |
| Condition·baselineAMP | 1.8159 | 0.0088 |
| Extroversion | 0.0189 | 0.0112 |

This table shows the standard normal regression of the mean amplitude of the SCRs $A_{i}$ on a number of independent and explanatory variables, as defined in Table S2. A new explanatory variable here is baselineAMP which is the mean amplitude of the SCRs in the baseline. The least squares parameter estimates are shown, together with the significance levels (P) for the t-tests of the null hypothesis that the corresponding parameter is zero. The result shows that *A* is significantly lower for HC than for VC and covaries positively with SCR rate, positively with baselineAMP, and positively with extroversion. The overall fit has $R^{2}=0.43$, (F = 5.5146, d.f. = 29, P = 0.002). The hypothesis that the residuals of the fit follow a normal distribution is not rejected by a two-tailed Kolmogorov-Smirnov test (P = 0.94).

Table S4 - Event Related Heart-rate in bpm for (a) VC and (b) HC in intervals Prior-shock and Reaction. *N* = 15 RR intervals were used for each segment

| **(a) VC** | **HR [bpm]** | | |
| --- | --- | --- | --- |
| **Subject** | **Prior-shock** | **Reaction** | **Difference** |
| 1 | 86.5700 | 89.4323 | 2.8623 |
| 2 | 81.1166 | 85.1981 | 4.0815 |
| 3 | 98.4615 | 100.3922 | 1.9306 |
| 4 | 68.7248 | 68.8789 | 0.1541 |
| 5 | 104.2365 | 105.6967 | 1.4602 |
| 6 | 94.7313 | 93.4550 | -1.2763 |
| 7 | 82.5806 | 82.6282 | 0.0476 |
| 8 | 62.6300 | 63.5649 | 0.9349 |
| 101 | 73.7069 | 74.9791 | 1.2721 |
| 102 | 53.2541 | 54.5441 | 1.2900 |
| 103 | 96.7777 | 97.4796 | 0.7019 |
| 104 | 64.7029 | 64.8394 | 0.1366 |
| 105 | 57.2067 | 56.8588 | -0.3479 |
| 106 | 60.1258 | 62.3575 | 2.2317 |
| 107 | 82.8990 | 85.5881 | 2.6891 |
| 109 | 67.2315 | 68.1693 | 0.9378 |
| 110 | 75.5852 | 77.3386 | 1.7534 |
| 111 | 88.8045 | 86.8322 | -1.9722 |
| 113 | 66.8657 | 68.3100 | 1.4444 |
| 301 | 86.9551 | 87.7535 | 0.7984 |
| 302 | 84.3294 | 83.9508 | -0.3786 |
| 303 | 65.9834 | 67.9861 | 2.0027 |
| 304 | 63.1449 | 62.2402 | -0.9047 |

The sign test for paired samples results in p=0.01.

| **(b) HC** | **HR [bpm]** | | |
| --- | --- | --- | --- |
| **Subject** | **Prior-shock** | **Reaction** | **Difference** |
| 401 | 84.1809 | 84.2633 | 0.0825 |
| 403 | 75.8385 | 74.8486 | -0.9899 |
| 405 | 69.2560 | 69.9203 | 0.6643 |
| 406 | 68.4732 | 69.7842 | 1.3110 |
| 407 | 96.4738 | 97.5902 | 1.1164 |
| 408 | 76.1204 | 74.3568 | -1.7635 |
| 409 | 70.9352 | 71.7039 | 0.7687 |
| 410 | 79.0734 | 79.0588 | -0.0145 |
| 411 | 78.0970 | 78.8559 | 0.7589 |
| 412 | 112.0876 | 110.5888 | -1.4987 |
| 413 | 85.5370 | 85.3503 | -0.1867 |

The sign test does not show a significant difference.

Table S5 - Event Related Heart-Rate Variability from *N* = 8 beats for (a) VC and (b) HC in intervals Prior-shock and Reaction.

| **(a) VC** | **STD (HRV) [ms]** | | |
| --- | --- | --- | --- |
| **Subject** | **Prior-shock** | **Reaction** | **Difference** |
| 1 | 10.6246 | 10.0472 | -0.5774 |
| 2 | 10.9637 | 10.1548 | -0.8089 |
| 3 | 5.9907 | 5.2447 | -0.7459 |
| 4 | 10.3043 | 10.6742 | 0.3699 |
| 5 | 8.6071 | 6.5633 | -2.0438 |
| 6 | 7.4835 | 6.5572 | -0.9263 |
| 7 | 12.9652 | 12.5913 | -0.3739 |
| 8 | 32.7493 | 23.9967 | -8.7526 |
| 101 | 10.0454 | 10.6213 | 0.5760 |
| 102 | 21.5062 | 30.1267 | 8.6205 |
| 103 | 7.4378 | 5.5867 | -1.8511 |
| 104 | 12.9243 | 12.6232 | -0.3011 |
| 105 | 18.0758 | 15.3492 | -2.7266 |
| 106 | 21.4083 | 20.6497 | -0.7586 |
| 107 | 10.0719 | 9.7737 | -0.2982 |
| 109 | 9.8766 | 11.0016 | 1.1250 |
| 110 | 6.2979 | 5.8025 | -0.4954 |
| 111 | 9.7898 | 9.6555 | -0.1343 |
| 113 | 15.9094 | 11.6284 | -4.2811 |
| 301 | 4.9968 | 4.7068 | -0.2900 |
| 302 | 8.2893 | 7.4237 | -0.8656 |
| 303 | 12.5475 | 10.1118 | -2.4357 |
| 304 | 19.0707 | 16.5004 | -2.5703 |

The sign test for paired samples results in p<0.01.

| **(b) HC** | **STD (HRV) [ms]** | | |
| --- | --- | --- | --- |
| **Subject** | **Prior-shock** | **Reaction** | **Difference** |
| 401 | 11.8167 | 8.8438 | -2.9729 |
| 403 | 20.1225 | 15.9356 | -4.1869 |
| 405 | 15.0064 | 15.9211 | 0.9147 |
| 406 | 8.2011 | 11.3732 | 3.1721 |
| 407 | 9.7441 | 7.6328 | -2.1113 |
| 408 | 8.4974 | 7.7446 | -0.7528 |
| 409 | 9.0380 | 8.7271 | -0.3109 |
| 410 | 27.8162 | 27.4412 | -0.3750 |
| 411 | 8.7731 | 9.3016 | 0.5285 |
| 412 | 6.8970 | 6.0294 | -0.8676 |
| 413 | 10.5554 | 8.9523 | -1.6031 |

The sign test does not show a significant difference.

Table S6 – Significance levels (P) for sign tests for differences between event related heart rates before and after the shocks for a range of RR Intervals *N*

| ***N*** | **P for VC** | **P for HC** |
| --- | --- | --- |
| 3 | 0.5235 | 1.0000 |
| 4 | 0.6776 | 0.5488 |
| 5 | 0.4049 | 0.5488 |
| 6 | 0.5235 | 0.5488 |
| 7 | 1.0000 | 0.0654 |
| 8 | 0.4049 | 1.0000 |
| 9 | 0.6776 | 1.0000 |
| 10 | 0.2100 | 1.0000 |
| 11 | 0.6776 | 1.0000 |
| 12 | 0.2100 | 1.0000 |
| 13 | 0.0347 | 1.0000 |
| 14 | 0.0106 | 1.0000 |
| 15 | 0.0106 | 1.0000 |
| 16 | 0.0931 | 0.2266 |
| 17 | 0.0931 | 0.5488 |
| 18 | 0.0931 | 0.5488 |
| 19 | 0.0931 | 0.2266 |
| 20 | 0.0347 | 0.5488 |
| 21 | 0.2100 | 1.0000 |
| 22 | 0.0347 | 1.0000 |
| 23 | 0.0347 | 1.0000 |
| 24 | 0.0347 | 0.2266 |
| 25 | 0.0347 | 0.0654 |
| 26 | 0.0931 | 0.5488 |
| 27 | 0.0931 | 1.0000 |
| 28 | 0.2100 | 1.0000 |
| 29 | 0.6776 | 0.5488 |
| 30 | 0.2863 | 1.0000 |

The table gives the P-values for paired sign tests for the difference in event related heart rate, prior to the shock and the reaction to the shock. The tests are over a range of RR intervals (Table S4 uses *N* = 15, for example). It can be seen that at the 5% level there are several values for *N* that result in significance in the VC, but none in the HC.

Table S7 – Significance levels (P) for sign tests for differences between event related heart rate variability before and after the shocks over a range of different numbers of beats, *N*

| ***N*** | **P for VC** | **P for HC** |
| --- | --- | --- |
| 3 | 0.4049 | 1.0000 |
| 4 | 0.2100 | 1.0000 |
| 5 | 0.6776 | 0.2266 |
| 6 | 0.0026 | 0.0654 |
| 7 | 0.0106 | 0.0654 |
| 8 | 0.0026 | 0.2266 |
| 9 | 0.0347 | 0.2266 |
| 10 | 0.0347 | 0.2266 |
| 11 | 0.0931 | 0.2266 |
| 12 | 0.4049 | 0.0654 |
| 13 | 0.6776 | 0.5488 |
| 14 | 0.6776 | 0.5488 |
| 15 | 1.0000 | 1.0000 |
| 16 | 1.0000 | 0.5488 |
| 17 | 1.0000 | 1.0000 |
| 18 | 1.0000 | 1.0000 |
| 19 | 1.0000 | 1.0000 |
| 20 | 0.6776 | 0.5488 |
| 21 | 0.4049 | 0.2266 |
| 22 | 0.6776 | 0.2266 |
| 23 | 0.6776 | 0.2266 |
| 24 | 0.6776 | 0.0654 |
| 25 | 0.6776 | 0.2266 |
| 26 | 1.0000 | 0.2266 |
| 27 | 1.0000 | 0.5488 |
| 28 | 1.0000 | 0.2266 |
| 29 | 1.0000 | 0.5488 |
| 30 | 1.0000 | 0.2266 |

The table gives the P-values for paired sign tests for the difference in event related heart rate variability, prior to the shock and the reaction to the shock. The tests are over a range of numbers of beats (Table SI5 uses *N* = 8, for example). It can be seen that at the 5% level there are several values for *N* that result in significance in the VC, but none in the HC.

# Movies

The video sequences show extracts from the experiment in the Visible Condition. Due to ethical constraints we are unable to supply the original video material of the participants in the actual experiments. These therefore show one of the authors in his first exposure to the experiment. They are for illustrative purposes only.

### MovieS1 (4.22MB)

This shows the events leading up to the 9^th^ shock, and also shows how the shock is administered with the machine.

### MovieS2 (4.54MB)

This shows the events leading up to the 6^th^ shock, the one where the Learner refuses to answer for the first time.

### MovieS3

### split into two files MovieS3a (9.78MB) and MovieS3b (8.21MB)

This includes the events at the final two questions.

### Movie S4 – available online

<http://www.cs.ucl.ac.uk/presencia/MilgramVR>

This shows the complete (VC) experiment. There are 7 clips in order, varying in size between 15 and 18MB.
