# Supplementary material for: A Virtual Reprise of the Stanley Milgram Obedience Experiments
Source: PLoS One. 2006 Dec 20;1(1):e39. doi: 10.1371/journal.pone.0000039 (PMC1762398; doi:10.1371/journal.pone.0000039)
Supplement: Figure S1 — Skin conductance waveform average around the shock times for the Hidden Condition. Event triggered average of 20 s segments of skin conductance waveform, the events being the times when buttons that gave an electric shock to the virtual character were pressed. The mean was calculated over each shock and each person in the HC (n = 220). Each waveform was first adjusted by subtracting the corresponding individual's mean SCL during the baseline period. For each participant a number of pseudo random shock times equal to the actual number for that person were generated. An average curve was formed like this 500 times, and these are shown as the many overlapping thinner curves. (0.04 MB DOC) [file pone.0000039.s002.doc]

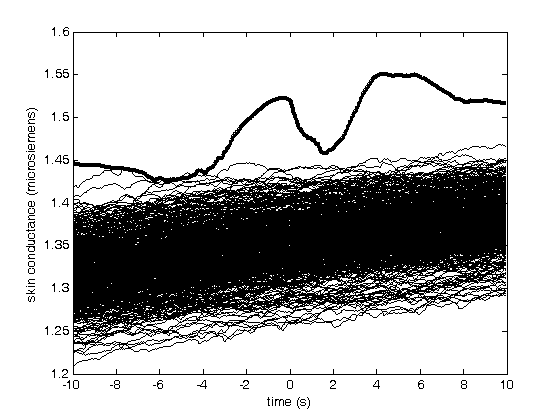


**Figure S1** Skin conductance waveform average around the shock times for the Hidden Condition. Event triggered average of 20s segments of skin conductance waveform, the events being the times when buttons that gave an electric shock to the virtual character were pressed. The mean was calculated over each shock and each person in the HC (n=220). Each waveform was first adjusted by subtracting the corresponding individual’s mean SCL during the baseline period. For each participant a number of pseudo random shock times equal to the actual number for that person were generated. An average curve was formed like this 500 times, and these are shown as the many overlapping thinner curves.
