# Supplementary material for: A Virtual Reprise of the Stanley Milgram Obedience Experiments
Source: PLoS One. 2006 Dec 20;1(1):e39. doi: 10.1371/journal.pone.0000039 (PMC1762398; doi:10.1371/journal.pone.0000039)
Supplement: Figure S2 — Comparison of skin conductance waveform averages for the VC (continuous curves) and HC (dashed curves). The mean waveforms are constructed as in Fig. 2 and Figure S1. All individuals have had their mean baseline SCL subtracted as before. 95% Normal (non-simultaneous) confidence intervals are shown. If we take any point in time then the individual values that went into making up the means at that point can be used to directly test the null hypothesis that the two samples could have come from the same population. For example, at time 0 (when the shock was given) the Wilcoxon rank sum test would reject the null hypothesis with P = 2.3×10^−20^. (0.03 MB DOC) [file pone.0000039.s003.doc]

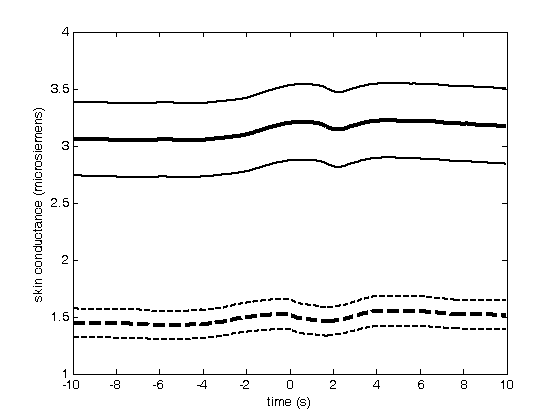


**Figure S2** Comparison of skin conductance waveform averages for the VC (continuous curves) and HC (dashed curves). The mean waveforms are constructed as in Fig. 2 and Figure S1. All individuals have had their mean baseline SCL subtracted as before. 95% Normal (non-simultaneous) confidence intervals are shown. If we take any point in time then the individual values that went into making up the means at that point can be used to directly test the null hypothesis that the two samples could have come from the same population. For example, at time 0 (when the shock was given) the Wilcoxon rank sum test would reject the null hypothesis with P = 2.3×10^-20^.
