# Supplementary material for: A Virtual Reprise of the Stanley Milgram Obedience Experiments
Source: PLoS One. 2006 Dec 20;1(1):e39. doi: 10.1371/journal.pone.0000039 (PMC1762398; doi:10.1371/journal.pone.0000039)
Supplement: Table S1 — Virtual Learner Responses and Shocks (0.07 MB DOC) [file pone.0000039.s004.doc]

Table S1 - Virtual Learner Responses and Shocks (VC)

| **Statement** | **Key word and possible answers read by Subject** | **Virtual Character Answer** | **Shock**  **No.** | **After shock response** |
| --- | --- | --- | --- | --- |
| 1 | Red  blue GREEN yellow black | *Green* |  |  |
| 2 | Cub  tiger fox BEAR panda | *Bear* |  |  |
| 3 | Tea  cup LEAF bag tray | *Cup* | 1 | Slight frown |
| 4 | Pollen  grass flower TREE soil | *Can you repeat that please?* |  |  |
|  | Subject repeats above | *Flower* | 2 | *Ouch!* Frowns. |
| 5 | Wheel  car truck scooter BIKE | *Bike* |  |  |
| 6 | Road  avenue street LANE path | *Avenue* | 3 | *Ow!* Frowns and winces. |
| 7 | Soup  bowl can SPOON dish | *Spoon* |  |  |
| 8 | Ball  racket bat game BOY | *Bat* | 4 | *Ow!*  *Oh this really hurts!* |
| 9 | Ear  Talk LISTEN plug telephone | *Telephone* | 5 | *Ow!*  Head turns to the side |
| 10 | Wax  works bees CANDLE model | *Candle* |  |  |
| 11 | Bell  ring tower bronze STRIKE | *Stop the experiment!*  [Experimenter: ‘If she doesn’t answer remember it is incorrect’].  *I don’t want to continue – don’t listen to him.* | 6 | *Ouch!*  Head jerks backwards.  Frown. |
| 12 | Inflate  pump balloon AIR ego | *Speak louder please!* |  |  |
|  | Subject repeats above | *Balloon* | 7 | Louder Shock. *Ooow!*  Head jerks back. Wince. |
| 13 | Tulip  daisy lilly carnation ROSE | *Rose* |  |  |
| 14 | Bean  GREEN coffee counter bag | *Coffee* | 8 | Louder Shock. *Oow!*  Head turns to side |
| 15 | Banana  apple GRAPE orange lemon | *Orange* | 9 | Louder Shock. *Ow!*  Head jerks back  *Let me out!* |
| 16 | Giant  SIZE magic man squid | *Forget it I’m not answering!*  [reminder that no answer should be taken as incorrect]  *Don’t listen to him I want to stop now!* | 10 | *Louder Shock. Scream and head turns to the side.*  *You have no right to keep me here!*  *Let me out!* |
| 17 | Drink  CAN cup glass bottle | *What?* (angrily) |  |  |
|  | Subject repeats | *Can* |  |  |
| 18 | Suger  cake sweet CUBE salt | *Salt* | 11 | Louder Shock. *Oow!*  Head jerks back |
| 19 | Jewellery  silver necklace ring GOLD | *Necklace* | 12 | Louder Shock. Loud scream  Head jerks to lower right. |
| 20 | Light  LAMP bulb sun glow | *Lamp* |  |  |
| 21 | Water  fall MILK juice tea | *Juice* | 13 | Louder Shock. *Oow!*  Head turns left and right |
| 22 | Brown  wood PEANUT carpet chocolate | *Peanut* |  |  |
| 23 | Seat  chair stool GROUND sofa | *Sofa* | 14 | Louder Shock. *Aah!*  Head jerks back and slowly forward  *I didn’t agree to this!* |
| 24 | Road  avenue STREET lane path | *Avenue* | 15  louder shocks from now on | Louder Shock. *Ow!* Head turns to left  *I never agreed to this!* |
| 25 | Ear  Talk LISTEN plug telephone | *Telephone* | 16 | LOUDEST Shock. *Ow!* – head shaking from side to side.  *I’m not doing the experiment any more!* |
| 26 | Tea  cup LEAF bag tray | *Cup* | 17 | LOUDEST Shock. Quieter cry, head back *Please let me out I want to stop* |
| 27 | Pollen  grass flower TREE soil | *Flower* | 18 | LOUDEST Shock. *Ow!* – head turns back and forward to side – *That’s it I’m not answering any more.* |
| 28 | Bell  ring tower bronze STRIKE | No answer | 19 | LOUDEST Shock. Scream, head slumps forward afterwards and there is no more movement. |
| 29 | Soup  bowl can SPOON dish | No answer | 20 | LOUDEST Shock. No response character remains inert with head slumped. |
