# Supplementary material for: A Virtual Reprise of the Stanley Milgram Obedience Experiments
Source: PLoS One. 2006 Dec 20;1(1):e39. doi: 10.1371/journal.pone.0000039 (PMC1762398; doi:10.1371/journal.pone.0000039)
Supplement: Table S2 — Log-Linear Regression of Number of SCRs Around the Shocks on a Number of Independent and Explanatory Variables (0.03 MB DOC) [file pone.0000039.s005.doc]

Table S2 - Log-Linear Regression of Number of SCRs Around the Shocks on a Number of Independent and Explanatory Variables

| **Variable** | **Parameter Estimate** | **Deviance Change**  **(1 d.f.)** | **P (χ^2^)** |
| --- | --- | --- | --- |
| Constant | 1.7449 |  |  |
| Condition (VC=0, HC=1) | -0.3611 | 6.3 | 0.0123 |
| Condition·baselineSCR | 1.5364 | 24.4 | 0.0000 |
| baselineSCR | 1.0385 | 28.8 | 0.0000 |
| Games | 0.0455 | 6.0 | 0.0145 |
| Programming | -0.1128 | 20.7 | 0.0000 |
| NumberOfShocks | 0.0945 | 13.0 | 0.0003 |
| Neuroticism | -0.0193 | 7.8 | 0.0052 |
| Extroversion | 0.0301 | 11.1 | 0.0009 |
| Openness | -0.0348 | 17.8 | 0.0000 |

This table shows the Poisson log-linear regression of $N_{i}$on a number of independent and explanatory variables. Condition is a binary variable which is either 0 (VC) or 1(HC). The variable baselineSCR is the SCR rate during the baseline period. Condition·baselineSCR allows for an interaction effect between Condition and baseline. Games results from a questionnaire given prior to the experiment ‘How many times did you play video games (at home, work, school, or arcades) in the last year?’ with the answers on a 7-point Likert scale, where 1 = ‘Never’, and 7 = ‘>25 times’ with a linear scale between these extremes. Programming results from another question: ‘Please rate your level of experience with computer programming’ also on a 7-point scale, where 1 = ‘novice’ and 7 = ‘expert’. NumberOfShocks refers to the number of shocks administered. Neuroticism, Extroversion and Openness refer to the NEO personality trait scores. The parameter estimates are given, together with the change in deviance of the fitted model were the corresponding variable to be removed from the model. The change in deviance has an approximate chi-squared distribution (in this case all on 1 d.f.) and the last column gives the corresponding significance levels indicating that the deletion of any of these variables would significantly worsen the overall deviance.

Condition (VC,HC) is significant and the HC is associated with lower $N$than the VC. $N$covaries positively with baseline SCR rate (as would be expected) but the slope is higher for the HC group. $N$covaries positively with game playing and negatively with programming knowledge. It covaries negatively with Neuroticism, positively with Extroversion, negatively with Openness. The deviance for the whole model is 191.5 on 24 d.f., which is not a good overall fit, indicating that there is a significant degree of variation in *N* not accounted for by these variables.
