# Supplementary material for: A Virtual Reprise of the Stanley Milgram Obedience Experiments
Source: PLoS One. 2006 Dec 20;1(1):e39. doi: 10.1371/journal.pone.0000039 (PMC1762398; doi:10.1371/journal.pone.0000039)
Supplement: Table S3 — Normal Regression of Mean Amplitude of SCRs Around the Shocks on a Number of Independent and Explanatory Variables (0.02 MB DOC) [file pone.0000039.s006.doc]

Table S3 - Normal Regression of Mean Amplitude of SCRs Around the Shocks on a Number of Independent and Explanatory Variables

| **Variable** | **Parameter Estimate** | **P (t-test)** |
| --- | --- | --- |
| Constant | -0.2962 | 0.1811 |
| Condition (VC=0; HC=1) | -0.2812 | 0.0254 |
| baselineSCR | 0.3147 | 0.0394 |
| Condition·baselineAMP | 1.8159 | 0.0088 |
| Extroversion | 0.0189 | 0.0112 |

This table shows the standard normal regression of the mean amplitude of the SCRs $A_{i}$ on a number of independent and explanatory variables, as defined in Table S2. A new explanatory variable here is baselineAMP which is the mean amplitude of the SCRs in the baseline. The least squares parameter estimates are shown, together with the significance levels (P) for the t-tests of the null hypothesis that the corresponding parameter is zero. The result shows that *A* is significantly lower for HC than for VC and covaries positively with SCR rate, positively with baselineAMP, and positively with extroversion. The overall fit has $R^{2}=0.43$, (F = 5.5146, d.f. = 29, P = 0.002). The hypothesis that the residuals of the fit follow a normal distribution is not rejected by a two-tailed Kolmogorov-Smirnov test (P = 0.94).
