# Supplementary material for: A Virtual Reprise of the Stanley Milgram Obedience Experiments
Source: PLoS One. 2006 Dec 20;1(1):e39. doi: 10.1371/journal.pone.0000039 (PMC1762398; doi:10.1371/journal.pone.0000039)
Supplement: Table S4 — Event Related Heart-rate in bpm for (a) VC and (b) HC in intervals Prior-shock and Reaction. N = 15 RR intervals were used for each segment (0.06 MB DOC) [file pone.0000039.s007.doc]

Table S4 - Event Related Heart-rate in bpm for (a) VC and (b) HC in intervals Prior-shock and Reaction. *N* = 15 RR intervals were used for each segment

| **(a) VC** | **HR [bpm]** | | |
| --- | --- | --- | --- |
| **Subject** | **Prior-shock** | **Reaction** | **Difference** |
| 1 | 86.5700 | 89.4323 | 2.8623 |
| 2 | 81.1166 | 85.1981 | 4.0815 |
| 3 | 98.4615 | 100.3922 | 1.9306 |
| 4 | 68.7248 | 68.8789 | 0.1541 |
| 5 | 104.2365 | 105.6967 | 1.4602 |
| 6 | 94.7313 | 93.4550 | -1.2763 |
| 7 | 82.5806 | 82.6282 | 0.0476 |
| 8 | 62.6300 | 63.5649 | 0.9349 |
| 101 | 73.7069 | 74.9791 | 1.2721 |
| 102 | 53.2541 | 54.5441 | 1.2900 |
| 103 | 96.7777 | 97.4796 | 0.7019 |
| 104 | 64.7029 | 64.8394 | 0.1366 |
| 105 | 57.2067 | 56.8588 | -0.3479 |
| 106 | 60.1258 | 62.3575 | 2.2317 |
| 107 | 82.8990 | 85.5881 | 2.6891 |
| 109 | 67.2315 | 68.1693 | 0.9378 |
| 110 | 75.5852 | 77.3386 | 1.7534 |
| 111 | 88.8045 | 86.8322 | -1.9722 |
| 113 | 66.8657 | 68.3100 | 1.4444 |
| 301 | 86.9551 | 87.7535 | 0.7984 |
| 302 | 84.3294 | 83.9508 | -0.3786 |
| 303 | 65.9834 | 67.9861 | 2.0027 |
| 304 | 63.1449 | 62.2402 | -0.9047 |

The sign test for paired samples results in p=0.01.

| **(b) HC** | **HR [bpm]** | | |
| --- | --- | --- | --- |
| **Subject** | **Prior-shock** | **Reaction** | **Difference** |
| 401 | 84.1809 | 84.2633 | 0.0825 |
| 403 | 75.8385 | 74.8486 | -0.9899 |
| 405 | 69.2560 | 69.9203 | 0.6643 |
| 406 | 68.4732 | 69.7842 | 1.3110 |
| 407 | 96.4738 | 97.5902 | 1.1164 |
| 408 | 76.1204 | 74.3568 | -1.7635 |
| 409 | 70.9352 | 71.7039 | 0.7687 |
| 410 | 79.0734 | 79.0588 | -0.0145 |
| 411 | 78.0970 | 78.8559 | 0.7589 |
| 412 | 112.0876 | 110.5888 | -1.4987 |
| 413 | 85.5370 | 85.3503 | -0.1867 |

The sign test does not show a significant difference.
