# Supplementary material for: A Virtual Reprise of the Stanley Milgram Obedience Experiments
Source: PLoS One. 2006 Dec 20;1(1):e39. doi: 10.1371/journal.pone.0000039 (PMC1762398; doi:10.1371/journal.pone.0000039)
Supplement: Table S5 — Event Related Heart-Rate Variability from N = 8 beats for (a) VC and (b) HC in intervals Prior-shock and Reaction (0.06 MB DOC) [file pone.0000039.s008.doc]

Table S5 - Event Related Heart-Rate Variability from *N* = 8 beats for (a) VC and (b) HC in intervals Prior-shock and Reaction.

| **(a) VC** | **STD (HRV) [ms]** | | |
| --- | --- | --- | --- |
| **Subject** | **Prior-shock** | **Reaction** | **Difference** |
| 1 | 10.6246 | 10.0472 | -0.5774 |
| 2 | 10.9637 | 10.1548 | -0.8089 |
| 3 | 5.9907 | 5.2447 | -0.7459 |
| 4 | 10.3043 | 10.6742 | 0.3699 |
| 5 | 8.6071 | 6.5633 | -2.0438 |
| 6 | 7.4835 | 6.5572 | -0.9263 |
| 7 | 12.9652 | 12.5913 | -0.3739 |
| 8 | 32.7493 | 23.9967 | -8.7526 |
| 101 | 10.0454 | 10.6213 | 0.5760 |
| 102 | 21.5062 | 30.1267 | 8.6205 |
| 103 | 7.4378 | 5.5867 | -1.8511 |
| 104 | 12.9243 | 12.6232 | -0.3011 |
| 105 | 18.0758 | 15.3492 | -2.7266 |
| 106 | 21.4083 | 20.6497 | -0.7586 |
| 107 | 10.0719 | 9.7737 | -0.2982 |
| 109 | 9.8766 | 11.0016 | 1.1250 |
| 110 | 6.2979 | 5.8025 | -0.4954 |
| 111 | 9.7898 | 9.6555 | -0.1343 |
| 113 | 15.9094 | 11.6284 | -4.2811 |
| 301 | 4.9968 | 4.7068 | -0.2900 |
| 302 | 8.2893 | 7.4237 | -0.8656 |
| 303 | 12.5475 | 10.1118 | -2.4357 |
| 304 | 19.0707 | 16.5004 | -2.5703 |

The sign test for paired samples results in p<0.01.

| **(b) HC** | **STD (HRV) [ms]** | | |
| --- | --- | --- | --- |
| **Subject** | **Prior-shock** | **Reaction** | **Difference** |
| 401 | 11.8167 | 8.8438 | -2.9729 |
| 403 | 20.1225 | 15.9356 | -4.1869 |
| 405 | 15.0064 | 15.9211 | 0.9147 |
| 406 | 8.2011 | 11.3732 | 3.1721 |
| 407 | 9.7441 | 7.6328 | -2.1113 |
| 408 | 8.4974 | 7.7446 | -0.7528 |
| 409 | 9.0380 | 8.7271 | -0.3109 |
| 410 | 27.8162 | 27.4412 | -0.3750 |
| 411 | 8.7731 | 9.3016 | 0.5285 |
| 412 | 6.8970 | 6.0294 | -0.8676 |
| 413 | 10.5554 | 8.9523 | -1.6031 |

The sign test does not show a significant difference.
