# Supplementary material for: A Virtual Reprise of the Stanley Milgram Obedience Experiments
Source: PLoS One. 2006 Dec 20;1(1):e39. doi: 10.1371/journal.pone.0000039 (PMC1762398; doi:10.1371/journal.pone.0000039)
Supplement: Table S7 — Significance levels (P) for sign tests for differences between event related heart rate variability before and after the shocks over a range of different numbers of beats, N (0.05 MB DOC) [file pone.0000039.s010.doc]

Table S7 – Significance levels (P) for sign tests for differences between event related heart rate variability before and after the shocks over a range of different numbers of beats, *N*

| ***N*** | **P for VC** | **P for HC** |
| --- | --- | --- |
| 3 | 0.4049 | 1.0000 |
| 4 | 0.2100 | 1.0000 |
| 5 | 0.6776 | 0.2266 |
| 6 | 0.0026 | 0.0654 |
| 7 | 0.0106 | 0.0654 |
| 8 | 0.0026 | 0.2266 |
| 9 | 0.0347 | 0.2266 |
| 10 | 0.0347 | 0.2266 |
| 11 | 0.0931 | 0.2266 |
| 12 | 0.4049 | 0.0654 |
| 13 | 0.6776 | 0.5488 |
| 14 | 0.6776 | 0.5488 |
| 15 | 1.0000 | 1.0000 |
| 16 | 1.0000 | 0.5488 |
| 17 | 1.0000 | 1.0000 |
| 18 | 1.0000 | 1.0000 |
| 19 | 1.0000 | 1.0000 |
| 20 | 0.6776 | 0.5488 |
| 21 | 0.4049 | 0.2266 |
| 22 | 0.6776 | 0.2266 |
| 23 | 0.6776 | 0.2266 |
| 24 | 0.6776 | 0.0654 |
| 25 | 0.6776 | 0.2266 |
| 26 | 1.0000 | 0.2266 |
| 27 | 1.0000 | 0.5488 |
| 28 | 1.0000 | 0.2266 |
| 29 | 1.0000 | 0.5488 |
| 30 | 1.0000 | 0.2266 |

The table gives the P-values for paired sign tests for the difference in event related heart rate variability, prior to the shock and the reaction to the shock. The tests are over a range of numbers of beats (Table SI5 uses *N* = 8, for example). It can be seen that at the 5% level there are several values for *N* that result in significance in the VC, but none in the HC.
